# Record of Meteorological Conditions at Buffalo, for Feb., 1856

**Published:** 1856-04

**Authors:** Sanford B. Hunt


					﻿ART. IV.—Record of Meteorological Conditions at Buffalo, for Feb.,
1856. By Sanford B. Hunt, M. D.
i 2 ® > i
□ qq	U
CO-	>• H
-.E.E«oS
4^ Cd t — r— *“
c co
SO C4—l j3 <o
4J O p d
tn O	g
.	•= a -b ~ *2 a
no	■— 2	*> =<-.
m	a o
Pi	©	03	a	S	« > .
2	gosS5--
a
a	g	<n	«	„
k	02	O h
£» 72
O -2 c
------= 2
e- p tuo-je ® s
02 r ? O O
P ~ jo 43 k>
•einpuadtuax I
jo	aiimw jrajuajijico	m	—	ct o	t^tcooo>nocoto	om	o	o	xf	co	ct
. notitr.duig jojcoco	. co to co to to to co to co to . to.	t£
j	’dniax ubojm ico	ci	^4	t— to	V	to	’ t—’	— ai ai to	id od	ci	ci	ci	to’	id
-  _________1 —1__________Cl_________I — Cl__________________________CM CM Cl CT fltl______—
©	.1 'a-In)” I .	CO	CO	co	co	co	co co co co	to co	to	co	to
Joclcnaj. ui;.)[^l t	co-r	V t—	V	to	—' od	cd o oi t~’	Vo	-4	cd	cd	V	cd
.-----------— —I__________Cl_________ I — Cl —_____CT CO d Cl Cl Cl________________________—
i UOUB.dOAa	do
2 jo ’dinar, id o od ci —	—' — id id id -4 cd O ci — o ci -4 ci cd 4
E _______ — —__________—■ Ct______I I Ct —I —	■—I	CT CT CT -I Ct — Cl___—
*■'•&>! •1
2 < 2? 'a-Itl?.<iru9xL,’ -J	ci ci	—' S id to to id cd —'	4 ci o' o id o' 4	4
H . 5_________—1 —'_________— C!_______I | Cl —' —< —•_________________Cl Cl Ct Cl Ct Cl C?__—
®	*	®	® -d	. ^' t£ .	. ^' .
g	= g = •=	CD CD	72	B W M
p	?	.	.	.	...
_________________O-tCT	Cl Cl	OOOCt-»CtOO	OOOOOOt — —_____
P b-tn>i3joi.iuv|g => o________________________________________________________oo_oooo	o	o o o_oooooooo_|_
i ’uoiin.dBAg .	............ ................................. .... —.
5	io	-diua r,	c-‘ 4 V V V ci o id	cd	ci	4	V ct od o'	ci	o' id ci	o	ci 4 4 oddridd	o
g	’_______,IJ	—< —._— CO —	—,	Cl	CO________cl	C!	— — —	CO	CO CO CO Ct CO d CO —_M
?	t—
.	, x .	............ .	.............. .... >.o
< 2c airii/WI3U V V jd o ci to' r-' ci to od cd ci ci —' o’ to 4 cd cd W o' ci od ci 4	—'
a________— —i__________—CO---- CO CO____Cl CO — — — COCOCOCO CO CO Ct CO CO_____________ct
7 g^S 4 iS	.^' . . f?	^ . .
CD CD CD CD CD CD CD CD	CD CD CD CD feSH
J 4 >d 4 4 rd ci o ci ci cd 4 — cicd ci id ci o -± o — ci_—‘ o ct ci —______________.
SpiV)[') Ji) j m y I	®
—---7--------------------——————-	'	57*
t uonnjlBxg	. .	.	. o ...........................
O 10 -daiar	r5 io u-j o	cri m -7 -7 r- m cr> cy o o to --	™
S	o r4
g Mni.dina.Ljg	o’ cd j 7 7 06	2? g^‘	%_!? ghS £ g 5
8-« ci	iTkIm-	-k?	. . I
-< S. s o’fe	CD CDCDCDD^r^r^
U	ci >d of' id cd ci ci ci ci cd -oi —‘ o -4 ci of ci o ci o o ci cd — — co ci —_
spnol3joi.iBv|220050ggsSg220222202002S2°22 2_____________________________________________L
o
>• ’upiljo-ni	2
j _____________________________________________—-------------------------------------------
a	ao oo 00 o ct 00	ctctt^ouotoofoooin.ao., — 01 o
•uioj'Bg jo nr	—; -d —> o	oooooooo-. 000000000 o
2ctctctwct«w__________co co ct ci ci ci co ct ct ci ct co ct co co co co	ct
I	awql-■	n 10	30	2 q 32 12 7 2 X ”2 2 _; -5 dr ct ct 4> ci ~A>	_
				

